# Differentiation of *Bacillus cereus* Species Based on Detected Unamplified Bacterial 16S rRNA by DNA Nanomachine

**DOI:** 10.21769/BioProtoc.5243

**Published:** 2025-03-20

**Authors:** Muhannad Ateiah, Erik R. Gandalipov, Aleksandr A. Rubel, Maria S. Rubel

**Affiliations:** 1 Laboratory of DNA-Nanosensor Diagnostics, ITMO University, Lomonosova 9, St. Petersburg, Russia; 2 Laboratory of Amyloid Biology, St. Petersburg State University, Universitetskaya enb. 7-9, St. Petersburg, Russia

**Keywords:** *B. cereus*, Detection of folded RNA, 10-23 DNAzyme, Amplification-free detection, 16S rRNA, Single nucleotide selectivity

## Abstract

Traditional approaches for the detection and differentiation of *Bacillus cereus* group species often face challenges due to the complexity of genetic discrimination between species. In this protocol, we propose a simple and straightforward assay based on the detected unamplified bacterial 16S rRNA by DNA nanomachine (DNM). The assay incorporates a universal fluorescent reporter and four DNA binding fragments, three of which are responsible for “opening up” the folded rRNA while the fourth strand is responsible for detecting single nucleotide variation (SNV) with high selectivity. The binding of the DNM to 16S rRNA results in the formation of the 10-23 DNAzyme catalytic core that cleaves the fluorescent reporter and produces a signal, which is amplified over time due to catalytic turnover. The developed biplex assay enables the detection of *B. thuringiensis* 16S rRNA and *B. mycoides* at fluorescein and Cy5 channels, respectively. The protocol offers two detection options: one utilizing extracted total RNA and the other involving crude cell lysate. The latter enables a fast and straightforward detection after 1.5 h with a hands-on time of ~15 min. The new protocol may simplify the analysis of biological RNA samples and might be useful for environmental monitoring as a simple and inexpensive alternative to amplification-based nucleic acid analysis.

Key features

• A sensitive and selective amplification-free biplex assay for differentiating *Bacillus thuringiensis* and *Bacillus mycoides* based on the 16S rRNA.

• A simple and inexpensive assay alternative to amplification-based nucleic acid analysis, useful in environmental monitoring applications.

• Adaptable for other challenging bacterial strains beyond *Bacillus cereu*s species.

## Background

The *Bacillus cereus* group is known for its diversity and widespread presence in the biosphere. While some species pose a significant risk as foodborne pathogens, others have agricultural or industrial applications [1]. For example, *Bacillus cereus (B. cereus*) causes two distinct forms of food poisoning [2]. In contrast, *B. thuringiensis* is widely used as an insecticidal agent and is generally considered non-pathogenic to humans, although it has been implicated in some foodborne outbreaks [3,4]. Species within the *B. cereus* group, including *B. cereus, B. thuringiensis, B. anthracis*, and *B. mycoides*, share extensive genomic and biochemical similarities, complicating their differentiation [5,6]. The true prevalence of *B. thuringiensis* in foodborne outbreaks remains unclear due to the inherent challenges in genetic differentiation within the *B. cereus* group. Species classification within this group often relies on phenotypic traits or the presence of species-specific genes such as *cry* genes [7], but this approach is hindered by polymorphisms [8] and the presence of *cry*-like genes in other *Bacillus* species [9] or even different genera [10]. The use of the 16S rRNA sequence as a reliable marker for the discrimination of *B. cereus* group members has failed in previous studies [11,12] due to high nucleotide conservation in the sequence among the species of this group. Therefore, developing detection strategies that are simple, affordable, and capable of distinguishing subtle genetic differences represents a critical step in microbial monitoring and public health management.

**Figure 1.**
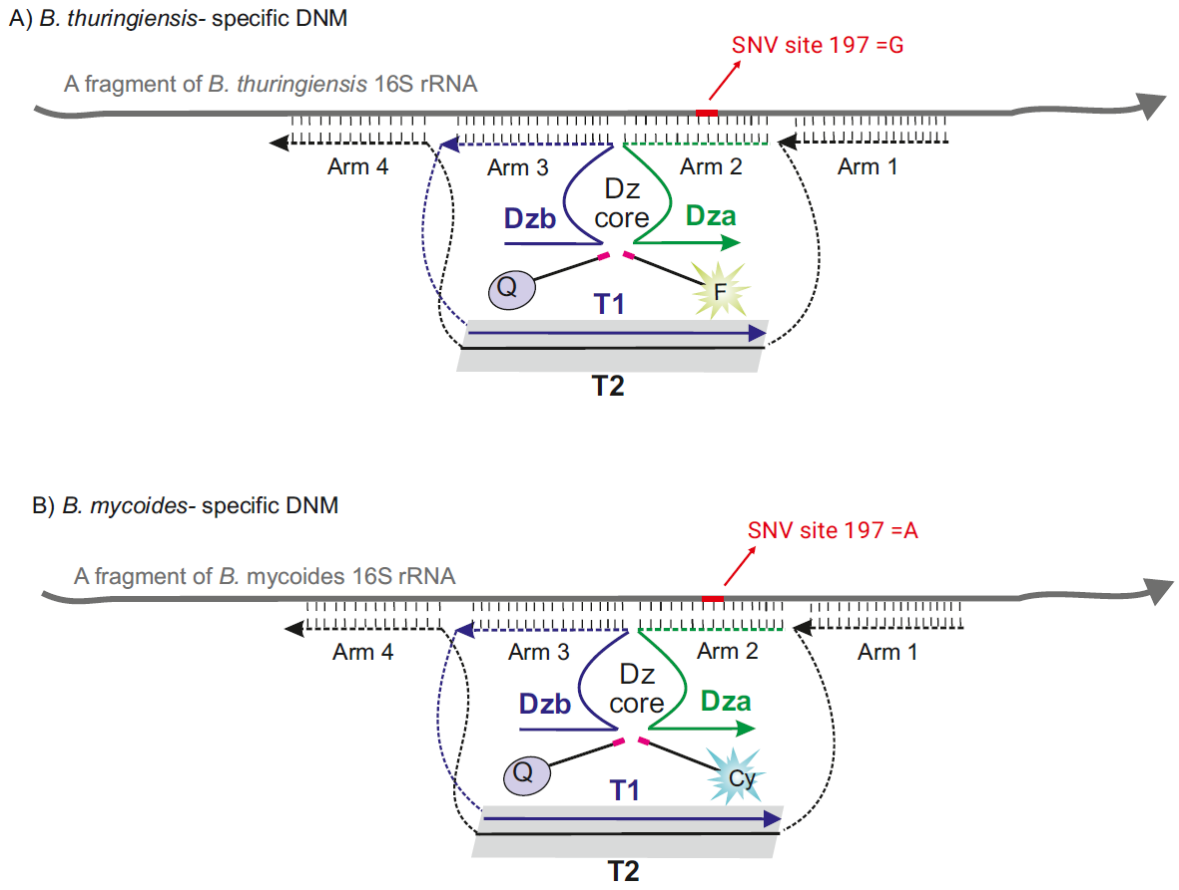
Design of the DNA nanomachine (DNM) used in the protocol. (A) Design of *B. thuringiensis*–specific DNM. The DNM has a separate arm (Dza) and three arms (Arm 1, Arm 3, and Arm 4) attached to the dsDNA scaffold. Arms 1 and 4 were designed to tightly bind 16S rRNA, thereby unfolding its secondary structure. *B. thuringiensis*–specific DNM cleaves F-sub, producing fluorescent output at 525 nm. (B) The design of *B. mycoides*–specific DNM is similar to that of *B. thuringiensis*, but it cleaves Cy-Sub, producing fluorescent output at 662 nm. *B. thuringiensis*–specific Dza and *B. mycoides*–specific Dza are complementary to a fragment of 16S rRNA containing two SNVs (192 and 197). SNV 197 is used to differentiate between *B. thuringiensis* and *B. mycoides*, whereas SNV 192 is used to differentiate between the two strains from *B. cereus* and *B. anthracis*.

Nucleic acid biosensors enhanced with functional nucleic acids have gained significant attention for a wide range of biomedical applications [13]. Among these, binary sensors equipped with a 10-23 DNAzyme core, known as binary RNA-cleaving DNAzyme 10-23 sensor (BiDz), are particularly prominent for molecular diagnostics [14,15]. The BiDz sensor incorporates two DNA strands, Dza and Dzb (Figure 1), which hybridize to a complementary fragment in the nucleic acid analyte and form a catalytically active Dz core. This facilitates a catalytic reaction that cleaves a reporter substrate labeled with a fluorophore and quencher, thus producing a fluorescent output.

Our continued efforts to refine the BiDz sensor for the detection of nucleic acids led to the BiDz-base nanostructure, the so-called “DNA nanomachine” (DNM) [16–18]. In addition to the binary sensor, this nanostructure includes (1) DNA facilitators, Arm 3 and 4 flanking the binary sensor (Figure 1), aiding in unwinding and invading into structured regions, and (2) a DNA tile that holds all the DNM components together in proximity and increases chances of the signal formation.

This protocol demonstrates the versatility of the multi-armed DNM approach for targeting and discriminating single-nucleotide variation (SNV) in natural 16S rRNA without prior amplification. The assay enables expedited quantification of rRNA content in cell cultures starting from whole cells. The DNM approach uses inexpensive reagents and equipment; it does not require perishable reagents (e.g., protein enzymes) and provides impressive selectivity toward SNV.

However, due to the high sequence similarity of the 16S rRNA among *B. cereus, B. thuringiensis, B. mycoides*, and *B. anthracis*, this protocol is specifically designed to detect and differentiate *B. thuringiensis* and *B. mycoides*. The SNV targeted by this assay is the only variation identified that allows discrimination between these species, as other SNVs in the 16S rRNA sequence are shared among multiple species, as shown in Table 1.

This protocol is less sensitive than PCR amplification-based methods, which remain the gold standard for nucleic acid detection in biological samples. Further development of DNM technology is anticipated to significantly lower the limit of detection (LOD) and reduce the assay time. Such improvements would not only enhance its competitiveness with state-of-the-art diagnostic techniques but also broaden its applicability to other difficult-to-discriminate pathogens.

**Table 1.** Comparison between the 16S rRNA SNV-containing fragments of *B. thuringiensis, B. mycoides*, and *B. cereus.*

Species	Sequence
*Bacillus thuringiensis*	ACAUUUUGAACUGCAU**G**G
*Bacillus mycoides*	AUAUUUUGAAC*U*GCAU**A**G
*Bacillus cereus*	ACAUUUUGAAC*C*GCAUGG

SNV 197 was subject to differentiation and is shown in bold. SNV 192 is shown in bold italic. SNV 182 is underlined [11].

## Materials and reagents

**Biological materials**

1. Cell cultures of *Bacillus cereus*

2. Cell cultures of *Bacillus thuringiensis*

3. Cell cultures of *Bacillus mycoides*

**Reagents**

1. Nuclease-free water (Invitrogen, catalog number: 1097704)

2. MgCl_2_ (AppliChem, catalog number: 13139)

3. NaCl (Vekton, catalog number: 557)

4. KCl (Carl Roth, catalog number: 178275)

5. HEPES (Amresco, catalog number: Am-O485)

6. Fine-powder acrylamide 4K (AA) (AppliChem, catalog number: A1090,0500)

7. Fine-powder bis-acrylamide (BA) (Molekula, catalog number: 2279795)

8. Ammonium persulfate (APS) (Carl Roth, catalog number: 9592.1)

9. TEMED (Molekula, catalog number: 68604730)

10. 10× TBE buffer (BioLabMix, catalog number: TBE-500)

11. RiboLock RNase inhibitor (40 U/μL) (Thermo Scientific, catalog number: EO0381)

12. Tryptone (Dia-M, catalog number: 1132GR500)

13. Yeast extract (Dia-M, catalog number: 210933)

14. 50 bp + DNA ladder (Evrogen, catalog number: NL003)

15. Ethidium bromide (BioLabMix, catalog number: EtBr-10)

16. Fine-powder agarose (Helicon, catalog number: SYS-Q0009-0.1)

17. 4× gel loading dye (Evrogen, catalog number: PB020)

18. Fluorescence substrate (DNA synthase, direct order, HPLC purification)

F-sub: 5′- AAG GTT(FAM) TCC TCg uCC CTG GGC A-BHQ1

Cy-sub: 5′- Cy5CAG CAC AAC Cg uCC CTG GGC A-BHQ-2

*Note: The stock solutions of the fluorescence substrate and the oligonucleotides mentioned below are prepared at a concentration of 100 μM. These stock solutions are provided in fine-powder form and should be diluted with nuclease-free water according to the manufacturer's instructions. The volume of nuclease-free water required will depend on the quantity in optical units specified by the individual placing the order.*

19. Oligonucleotides (Evrogen, direct order standard desalting purification):

**T1_thu:** 5′- TGC CCA GGGA *GG CTA GCT* TCA AAA TGT TAT CCG GTA /HEG/ **ACA CTT AGG ACT GCG AGA CCG ATG GTC AAG TCAC**

**T2_thu:** 5′- GTA AGT GAC AGC CGA AGC CGC CTT T /HEG/ **GTG ACT TGA CCA TCG GTC TCG CAG TCC TAA GTG T** /HEG/ TTA GCC CTG GTT TCC CGG AGT TAT CCC AG

**Dza_thu:** 5′- TCG AAC CAT GCA GT *ACAACGA* GAGGAAACCTT

**Analyte_thu:** 5′- TAA GAC TGG GAT AAC TCC GGG AAA CCG GGG CTA ATA CCG GAT AAC ATT TTG AAC TGC ATG GTT CGA AAT TGA AAG GCG GCT TCG GCT GTC ACT T

**T1_myc:** 5′- TGC CCA GGGA *GG CTA GCT* TCA AAA TAT TAT CCG GTA TT /HEG/ **AGT GCA ATG CCA GAC TTA GTA CCG ATC GGA TAA CCG TT**

**T2_myc:** 5′- AAG TGA CAG CCG AAG CCG CCT TTC /HEG/ **AAC GGT TAT CCG ATC GGT ACT AAG TCT GGC ATT GCA CT**/HEG/ AGC CCC GGT TTC CCG GAG TTA TCC CAG TCT TA

**Dza_myc:** 5′- ATT TCG AAC TAT GCA GT *ACAACGAG* GTTGTGCTG

**Analyte_myc:** 5′- TAA GAC TGG GAT AAC TCC GGG AAA CCG GGG CTA ATA CCG GAT AAT ATT TTG AAC TGC ATA GTT CGA AAT TGA AAG GCG GCT TCG GCT GTC ACT T

*Note: Nucleotides constituting the catalytic core of 10–23 deoxyribozymes are shown in italics. Nucleotides of the Dz strands that are complementary to the reporter substrate are underlined. Ribonucleotides are in lowercase. FAM: 6-carboxyfluorescein; BHQ1: Black Hole quencher 1; Cy5: Cyanine 5; BHQ2: Black Hole quencher 2; HEG: hexaethylene glycol. Tile sequence is in bold.*

**Solutions**

1. Col buffer (see Recipes)

2. 1 μM *B. thuringiensis*–specific DNM stock (see Recipes)

3. 1 μM *B. mycoides*–specific DNM stock (see Recipes)

4. 1× TBE buffer (see Recipes)

5. 0.9% NaCl (see Recipes)

6. Lysogeny (LB) broth (see Recipes)

7. 40% acrylamide:bis-acrylamide (AA:BA) (see Recipes)

8. 10% APS (see Recipes)

**Recipes**

**1. Col buffer**

**Table:** 

Reagent	Final concentration	Quantity or Volume
MgCl_2_ 1 M	200 mM	10 mL
KCl 1 M	150 mM	7.5 mL
HEPES 1 M	50 mM	2.5 mL
NaCl 1 M	15 mM	0.75 mL
Nuclease-free water	n/a	29.25 mL
Total		50 mL

**2. 1 μM *B. thuringiensis*–specific DNM stock**

**Table:** 

Reagent	Final concentration	Quantity or Volume
T1_thu 10 μM	1 μM	10 μL
T2_thu 10 μM	1 μM	10 μL
Col buffer	n/a	80 μL
Total		100 μL

**3. 1 μM *B. mycoides*–specific DNM stock**

**Table:** 

Reagent	Final concentration	Quantity or Volume
T1_myc 10 μM	1 μM	10 μL
T2_myc 10 μM	1 μM	10 μL
Col buffer	n/a	80 μL
Total		100 μL

**4. 1× TBE buffer**

**Table:** 

Reagent	Final concentration	Quantity or Volume
10× TBE buffer	1×	200 mL
Deionized water	n/a	1,800 mL
Total		2,000 mL

**5. 0.9% NaCl**

**Table:** 

Reagent	Final concentration	Quantity or Volume
NaCl	0.9%	0.9 g
Deionized water	n/a	100 mL
Total		100 mL

**6. Lysogeny (LB) broth**

**Table:** 

Reagent	Final concentration	Quantity or Volume
Tryptone	20 g/L	2 g
Yeast extract	10 g/L	1 g
NaCl	20 g/L	2 g
Deionized water	n/a	190.7
Total		200 mL

**7. 40% acrylamide:bis-acrylamide (AA:BA)**

**Table:** 

Reagent	Final concentration	Quantity or Volume
Acrylamide	38.96%	38.96 g
Bis-acrylamide	1.04%	1.04 g
Deionized water	n/a	100 mL
Total		100 mL

**8. 10% APS**

**Table:** 

Reagent	Final concentration	Quantity or Volume
APS	10%	10 g
Deionized water	n/a	100 mL
Total		100 mL

**Laboratory supplies**

1. Costar 96-well black polystyrene plate (Corning, catalog number: COS3915)

2. 1–10 μL pipette (Kirgen, catalog number: KG-Pro10)

3. 2–20 μL pipette (Kirgen, catalog number: KG-Pro20)

4. 20–200 μL pipette (Kirgen, catalog number: KG-Pro200)

5. 100–1,000 μL pipette (Kirgen, catalog number: KG-Pro1000)

6. Pipette 10 μL tips (Kirgen, catalog number: KG1111-L)

7. Pipette 200 μL tips (Kirgen, catalog number: KG1212-L)

8. Pipette 1,000 μL tips (Kirgen, catalog number: KG1636)

9. 1.5 mL microcentrifuge tube (Biofil, catalog number: CFT011015)

10. 15 mL centrifuge tube (Biofil, catalog number: CFT011150)

11. Sealing film for PCR plates (Sovtech, catalog number: P-502)

12. Parafilm^®^ M sealing film (Sigma-Aldrich, catalog number: HS234526C)

## Equipment

1. Mini Protean Tetra Cell (Bio-Rad, catalog number: 1658001EDU)

2. PowerPac^TM^ basic power supply (Bio-Rad, catalog number: 1645050)

3. Spark multimode microplate reader (Tecan, model: Spark-10M)

4. Water bath (LOIP, model: LB-140)

5. ChemiDoc^TM^ Touch Gel imaging system (Bio-Rad, catalog number: 1708370)

6. PCR workstation (Lamsystems, model: 2E-F.002-10)

7. Implen NanoPhotometer^TM^ N60 UV/V spectrophotometer (Implen, catalog number: 15715815)

8. ES 20 orbital shaker incubator (Biosan, catalog number: BS-010111-AAA)

9. Spectrophotometer UV-3000 with cuvettes (Promecolab, catalog number: 63493-16.3000UF)

10. RiOs-DI^®^ 3UV water purification system (Merck Millipore, model: ZRDSVP3WW)

## Software and datasets

1. UNAFold Web server

2. Basic Local Alignment Search Tool (BLAST) search tool

3. GraphPad Prism v. 9.3.1

4. CorelDRAW Graphics Suite 2024

## Procedure

**A. DNM design**

1. Select a unique region within the genome of interest or identify a region containing an SNV that is relevant to the targeted pathogen using the BLAST tool.

2. Analyze the secondary structure of the ssDNA fragment using the UNAFold Web Server:

a. Open the UNAFold Web Server, navigate to the *mFold* tab, then select *Applications*, and choose the *DNA Folding Form*.

b. Paste the sequence of the target region or upload the FASTA file into the provided input box.

c. Navigate to the *folding temperature* and choose 55 °C.

d. Navigate to the *Ionic Conditions* section and set [Mg++] to 200 mM and [Na+] to 215 mM.

e. Retain the default settings for all other parameters, as no further adjustments are needed.

f. Click the *Fold DNA* button to initiate the folding process.

g. Select a stable hairpin structure, ensuring that the DNM’s binding site is located within unstructured regions or, alternatively, on the loops of hairpin structures. This placement enhances the binding strength of the target.

3. If the targeted analyte site is folded into a stem-loop structure, position Arms 2 and 3 as follows:

• Arm 2: Design it to bind to one side of the stem, the loop, and 3–5 nucleotides on the second side of the stem. If a specific SNV is targeted, position Arm 2 such that the SNV is located at the center of Arm 2, ensuring optimal binding and selectivity for the target sequence.

• Arm 3: Design it to bind to a fragment on the second side of the stem.

4. Ensure that the melting temperature (Tm) of Arm 2 is at least 1–3 °C above the reaction temperature (55 °C), as it is responsible for the selectivity of the recognition, and the Tm of Arm 1, Arm 3, and Arm 4 is above 65 °C to ensure high binding strength:

a. Open the UNAFold Web Server, navigate to the *DINAMelt* tab, select *Applications*, and choose the *Two State Melting* hybridization.

b. Paste the sequence of the Arm in the first input box and the complementary sequence in the second input box.

c. Navigate down and set the following parameters:

• DNA at 55 °C.

• [Na+] to 215 mM.

• [Mg++] to 100 mM.

• Sequence type: 0.02 μM.

d. Click the *submit* button to calculate the melting temperature.

5. Combine the sequence of Arm 2 with the 10–23 deoxyribozyme core and the F-sub binding fragment:

Construct the Dza strand by starting with the Arm 2 sequence at the 5′ end, followed by the sequence of one-half of the 10–23 deoxyribozyme catalytic core, and concluding with the F-sub binding fragment for *B. thuringiensis* or the Cy-sub binding fragment sequence for *B. mycoides* at the 3′ end. This specific arrangement ensures proper functionality and structural alignment of the Dza strand.

6. Analyze the secondary structure of the designed Dza strands using the UNAFold Web Server as in steps A2a–f. Folding ΔG should not be lower than -4 kcal/mol. Try to avoid long GC-rich sequences.

7. Combine the sequence of Arm 3 with the 10–23 deoxyribozyme Core, the F-sub binding fragment, and the tile sequence:

• Construct the T1 strand by starting with the F-sub binding fragment sequence for *B. thuringiensis* or the Cy-sub binding fragment sequence for *B. mycoides* at the 5′ end, followed by the sequence of the second half of the 10–23 deoxyribozyme catalytic core, then the Arm 3 sequence and the HEG linker, and concluding with the tile sequence at the 3′ end. This specific arrangement ensures proper functionality and structural alignment of the T1 strand.

• You may use the provided tile sequence or, alternatively, create a random sequence for the DNA tile fragment that is the same length as Arm 2 and Arm 3. Ensure that the Tm of the tile sequence is above 65 °C and avoid sequences that are excessively enriched in GC content.

8. Analyze the secondary structure of the designed T1 strands using the UNAFold Web Server as in steps A2af. Folding ΔG should not be lower than -4 kcal/mol. Try to avoid long GC-rich sequences. If the secondary structure is too stable, introduce a point mutation to the Arm to reduce folding stability by increasing the ΔG. Preferably, put the mutation closer toward the ends of the arms, except those forming the core.

9. Combine the sequence of Arm 1 and Arm 4 with the complementary sequence of the tile fragment in T1: Construct the T2 strand by starting with the Arm 1 sequence at the 5′ end, followed by the HEG linker, then the complementary sequence of the tile fragment in T1 and the HEG linker, and concluding with the Arm 4 sequence at the 3′ end. This specific arrangement ensures proper functionality and structural alignment of the T2 strand.

10. Analyze the secondary structure of the designed T2 strands using the UNAFold Web Server as in steps A2af. Folding ΔG should not be lower than -4 kcal/mol. Try to avoid long GC-rich sequences.

• If the secondary structure of the designed strand is too stable, consider introducing mutations to Arm 1 and/or Arm 4 to reduce the folding stability by increasing the ΔG.

• Alternatively, to reduce stability, you can position Arm 1 2–3 nucleotides away from Arm 2 and/or place Arm 4 2–3 nucleotides away from Arm 3. These adjustments can help fine-tune the secondary structure for optimal performance.

11. Draw the DNM structure using CorelDRAW Graphics or any available vector graphics software. Verify accuracy in 5′→3′ directions for each strand.

12. Order the sequences from a reliable commercial vendor.

*Note: The sequence of the catalytic core, the fluorescence-substrate binding fragment, and the tile are fixed components of the DNM. The sequences of Arm 1, Arm 2, Arm 3, and Arm 4 are modifiable and change for each new target to enable specific binding to the nucleic acid region of interest. Additionally, the design of the DNM can be adapted from a biplex to a multiplex system to target more than two analytes. This adaptation requires only a new fluorogenic substrate and modifications to the substrate-binding fragment.*

**B. Assembly of DNM**

1. Prepare the 10 μM stock solutions of T1_thu, T2thu, T1_myc, and T2_myc using nuclease-free water in a PCR workstation and store them at -20 °C until use.

2. Completely thaw the oligonucleotide solutions before using. Gently mix by tapping (do not intensively vortex). Spin down the solution using a microcentrifuge.

3. Prepare the 1 μM *B. thuringiensis*–specific DNM aliquot (see Recipes) in a 1.5 mL microcentrifuge tube in the PCR workstation.

4. Prepare the 1 μM *B. mycoides*–specific DNM aliquot (see Recipes) in a 1.5 mL microcentrifuge tube in the PCR workstation.

5. Mix the tube gently and spin down the solution.

6. Wrap the lid of the closed tube with parafilm. Alternatively, use screw cap tubes.

7. Incubate the tubes in a 500 mL beaker with boiling water for 2 min and then turn the heater off and let the temperature passively cool down overnight.

8. Prepare the 12% native PAGE: Mix 1 mL of 10× TBE buffer, 3 mL of 40% AA:BA, MQ water up to 10 mL, 50 μL of 10% APS, and 5 μL of TEMED; move the mixture to the gel cassette and let it polymerize.

9. Mix 1 μL of each sample—including the assembled DNMs (*B. thuringiensis*–specific DNM and *B. mycoides*–specific DNM) and their individual components (T1_thu, T2_thu, T1_myc, and T2_myc)—with 1 μL of 4× loading dye. Load each mixture into separate wells of the gel.

10. Put the cassette into the chamber, fill it with 1× TBE buffer, and let the gel run for 90 min at 80 V.

11. Dye the gel with ethidium bromide and visualize it using a gel-documenting system.

**C. Characterization of DNM**

1. Prepare 160 μL of 200 nM F-sub in Col buffer in a 1.5 mL microcentrifuge tube. This is used to assess the basic background of the substrate stability.

2. Prepare seven aliquots of 160 μL, each containing 20 nM of preassembled *B. thuringiensis*–specific DNM, 20 nM of *B. thuringiensis*–specific Dza, and 200 nM of F-sub in reaction buffer in 1.5 mL microcentrifuge tubes. Out of the seven tubes, keep tube 1 as the blank background to assess the spontaneous assembly of the DNAzyme core.

3. Add the *B. thuringiensis*–specific analyte in tubes 2–7 at the final concentrations of 1, 10, 100, 200, 500, and 1,000 pM.

4. Gently mix, spin down the tubes, and divide the solutions into three 50 μL portions into the black 96-well plate.

5. Prepare 160 μL of 200 nM Cy-sub in reaction buffer in a 1.5 mL microcentrifuge tube. This is used to assess the basic background of the substrate stability.

6. Repeat steps C2–4 using 20 nM *B. mycoides*–specific DNM, 15 nM *B. mycoides*–specific Dza, 200 nM Cy-sub, and concentrations in the range of 1–1,000 pM of *B. mycoides*–specific analyte.

7. Seal the plate with an optically transparent film.

8. Incubate the plate in a water bath or any heating chamber at 55 °C for 1 h. Spin down the plate.

9. Measure fluorescence at 525 nm (λex = 495 nm) for F-sub in the *B. thuringiensis*. Calculate the mean and standard deviation for each point.

10. Measure fluorescence at 662 nm (λex = 617 nm) for Cy-sub in the *B. mycoides*. Calculate the mean and standard deviation for each point.

**D. Extraction of total RNA**

1. Use the protocol developed by Oh and So [19] to extract total RNA from the cell culture of *B. thuringiensis, B. mycoides*, and *B. cereus* or, alternatively, use any available protocol for extraction of total RNA from gram-positive bacteria. Assess the RNA quality via agarose gel electrophoresis.

**E. Detection of the extracted 16S rRNA using the DNM in extracted total RNA**

1. Measure the concentration of total RNA extracted from *B. thuringiensis, B. mycoides*, and *B. cereus* at a wavelength of 260 nm using the spectrophotometer.

2. Prepare 160 μL of 200 nM F-sub in reaction buffer in a 1.5 mL microcentrifuge tube.

3. Prepare nine aliquots of 160 μL, each containing 20 nM of preassembled *B. thuringiensis*–specific DNM, 20 nM of *B. thuringiensis*–specific Dza, and 200 nM of F-sub in reaction buffer in 1.5 mL microcentrifuge tubes. Out of the seven tubes, keep tube 1 as the blank background.

4. Add the *B. thuringiensis* total RNA to tubes 2–7 at the final concentrations of 1, 2, 4, 6, 8, and 10 ng/μL.

5. Add the *B. cereus* and *B. mycoides* total RNA to tubes 8 and 9, respectively, at the final concentrations of 10 ng/μL.

6. Gently mix, spin down the tubes, and divide the solutions into three 50 μL portions into the black 96-well plate.

7. Prepare 160 μL of 200 nM Cy-sub in reaction buffer in a 1.5 mL microcentrifuge tube.

8. Repeat steps E3–5 using 20 nM *B. mycoides*–specific DNM, 15 nM *B. mycoides*–specific Dza, 200 nM Cy-sub, and concentrations in the range of 1–10 ng/μL of *B. mycoides* total RNA in the same reaction buffer.

9. Add the *B. cereus* and *B. thuringiensis* total RNA in tubes 8 and 9, respectively, at the final concentrations of 10 ng/μL.

10. Gently mix, spin down the tubes, and divide the solutions into three 50 μL portions into the black 96-well plate.

11. Seal the plate with an optically transparent film.

12. Incubate the plate in a water bath at 55 °C for 1 h. Spin down the plate.

13. Measure fluorescence at 525 nm (λex = 495 nm) for F-sub. Calculate the mean and standard deviation for each point.

14. Measure fluorescence at 662 nm (λex = 617 nm) for Cy-sub. Calculate the mean and standard deviation for each point.

**F. Detection of the 16S rRNA using the DNM in crude cell lysates**

1. Inoculate 5 mL of freshly autoclaved LB broth with *B. thuringiensis, B. mycoides*, and *B. cereus* in separate 15 mL centrifuge tubes. Cultivate *B. thuringiensis* and *B. cereus* at 37 °C with shaking under 250 rpm for 16 h, and *B. mycoides* at 30 °C without shaking for 16 h.

2. Transfer 1 mL of the *B. thuringiensis, B. mycoides*, and *B. cereus* cell cultures into separate 1.5 mL microcentrifuge tubes and measure the OD_600_ using a spectrophotometer.

3. Dilute the cell cultures of *B. thuringiensis, B. mycoides*, and *B. cereus* to an OD_600_ of 1 using sterile 0.9% NaCl. This corresponds to approximately 10^7^ CFU/mL for *B. thuringiensis*, 10^8^ CFU/mL for *B. mycoides*, and 10^9^ CFU/mL for *B. cereus.*

4. Transfer 100 μL of the *B. thuringiensis* cell cultures to a 1.5 mL microcentrifuge tube containing 900 μL of 0.9% NaCl to create a 10^6^ CFU/mL dilution.

5. Mix thoroughly and repeat this process to make serial dilutions from 10^5^ CFU/mL to ~0 CFU/mL.

6. Transfer 10 μL of *B. mycoides* cell cultures to a 1.5 mL microcentrifuge tube containing 990 μL of 0.9% NaCl to create a 10^6^ CFU/mL dilution.

7. Transfer 10 μL of *B. cereus* cell cultures to a 1.5 mL microcentrifuge tube containing 990 μL of 0.9% NaCl to create a 10^7^ CFU/mL dilution. Then, make additional dilutions to reach 10^6^ CFU/mL.

8. Collect the *B. thuringiensis, B. mycoides*, and *B. cereus* by centrifugation at 900× *g* for 5 min for each tube in the serial dilutions.

9. Discard the supernatant from each tube and resuspend the pellet in 160 μL of the reaction buffer containing *B. thuringiensis*–specific DNM and Dza at a concentration of 20 nM.

10. Heat the mixture at 95 °C for 5 min and then leave it to cool down at room temperature for 5 min.

11. Centrifuge at 900× *g* for 3 min. Transfer the supernatant (~150 μL) from each serial dilution into clean 1.5 mL microcentrifuge tubes. Add 3.75 μL of RNase inhibitor to each tube to achieve the recommended final concentration of 1 U/μL. Subsequently, add the F-sub substrate to a final concentration of 200 nM.

12. Repeat steps F4–11 for *B. mycoides* using the reaction buffer containing 20 nM *B. mycoides*–specific DNM, 15 nM *B. mycoides*–specific Dza, and 200 nM Cy-sub.

*Note: When testing* B. mycoides*–specific DNM in step F6, take 1 mL of* B. thuringiensis *cell cultures directly without further diluting.*

13. Spin down the tubes and incubate at 55 °C for 1h.

14. Gently mix, spin down the tubes, and divide the solutions into three 50 μL portions into the black 96-well plate.

15. Measure fluorescence at 525 nm (λex = 495 nm) for F-sub.

16. Measure fluorescence at 662 nm (λex = 617 nm) for Cy-sub.

## Data analysis

**Figure 2.**
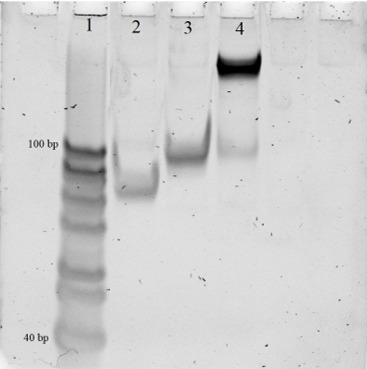
Analysis of DNA nanomachine (DNM) associations in 12% native PAGE gel. 1: 20–100 bp ladder; 2: T1_thur strand; 3: T2_thu strand; 4: Assembled DNM.

**Figure 3.**
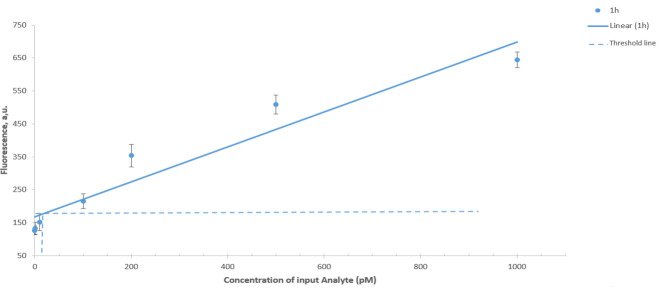
Limit of detection of synthetic DNA after 60 min of incubation at 55 °C. Horizontal lines stand for the threshold, and vertical lines correspond to the limit of detection (LOD). The signal was read at the FAM channel. Average values of three independent measurements are presented with one standard deviation.

1. The assembled DNM complex is evaluated for correct size and homogeneity using native PAGE (Figure 2). In Lane 4, the assembled DNM complex should appear as a single low-mobility band, indicating correct assembly. In Lane 3, one of the individual DNM strands (T2_thu) is loaded and should appear as a distinct band. In Lane 2, the other individual DNM strand (T1_thu) is loaded and should also appear as a distinct band. If the DNM is incorrectly assembled, Lane 4 will display two or three bold bands instead of a single band, indicating incomplete or incorrect assembly of the complex.

2. Two control values are measured: the pre-assembled DNM fragments and Dzb without the analyte (blank), and the background fluorescence from the F-sub only (basic).

3. To confirm the detection of the 16S rRNA by the DNM, the fluorescent signal of the assembled DNM with the analyte (synthetic or 16S rRNA) is compared with the negative control that does not contain any analyte added.

4. Signal-to-blank ratio (a ratio between the mean relative fluorescence units of the wells containing the analyte and the wells with no analyte) should be no less than 1.5 units to validate successful detection.

5. The blank-to-basic ratio (fluorescence of DNM response in the absence of analyte divided by that of F-sub-only control) should be in the range of 1.1–1.5.

6. Each concentration point is tested in triplicate (50 μL per replicate), requiring a total volume of 160 μL per concentration (including a 10 μL buffer excess to account for pipetting error).

7. Upload the relative fluorescence units to an Excel spreadsheet and prepare a scatter plot with a line to visualize the linear dependence between analyte concentration and fluorescence signal. Plot the analyte concentrations on the x-axis and the corresponding fluorescence measurements on the y-axis. Ensure the graph includes error bars representing standard deviations.

8. The limit of detection (LOD) is quantified as an intersection of the trendline and three standard deviations above the mean blank (fluorescence of DNM response in the absence of analyte) (Figure 3) [20]. The LOD is calculated using the following formula:

$$LOD\;=\;\frac{3\;\times \;\sigma b}{m}$$

where *σb* represents the standard deviation of the negative control and *m* represents the slope of the calibration curve.

9. Statistical analysis should be performed with three independent experimental repetitions ensuring that the standard deviation between the repeats is less than 0.5.

10. The fluorescent response of the DNM with a nonspecific target (e.g., *B. thuringiensis*–specific DNM tested with *B. cereus* or *B. mycoides* 16S rRNA) should be close to the background level observed in the absence of analyte to confirm accurate species differentiation.

## Validation of protocol

This protocol or parts of it has been used and validated in the following research article(s):

• Ateiah et al. [21]. DNA Nanomachine (DNM) Biplex Assay for Differentiating *Bacillus cereus* Species. *Int. J. Mol. Sci.* Limit of Detection of DNM with extracted total RNA: Figure S8, Limit of detection of DNM with crude cell lysate: Figure S9 and S10.

• Filatov et al. [22]. DNAzyme 10-23 – Based Nanomachines for Nucleic Acid Recognition. *J. Vis. Exp*. Assembly of DNM: Figure 2A, Characterization of DNM using synthetic DNA analyte: Figure 2B.

## General notes and troubleshooting

**General notes**

1. Perform the bacterial inoculation at sterile conditions according to the handling rules of the region.

2. Separate the experimental areas of the DNMs and the bacterial inoculations.

3. In case of changing the RNA extraction protocol or crude lysate preparation protocol, assess the quality of the cell disruption via microscopy before subjecting it to further procedures.

4. Do not heat the F-sub, the Cy-sub, or any mixture containing them over 65 °C.

5. Do not use concentrations of magnesium higher than 200 nM since high levels of magnesium ions promote spontaneous F-sub cleavage.

**Troubleshooting**

Problem 1: High fluorescent signal of F-sub only or DNM in the absence of the analyte.

Possible cause: Low quality of synthesis and purification of oligonucleotides.

Solution A: We suggest that the stability and purity of every oligonucleotide supply should be verified by native PAGE. The DNM method is sensitive to the quality of the oligonucleotide; therefore, the oligonucleotides should be carefully purified.

Solution B: Request the commercial supplier to perform additional purification steps for every oligonucleotide. Change the supplier in case of further issues.

Problem 2: Incorrect assembly of DNM.

Possible cause: The dsDNA fragments within the DNM have not been formed.

Solution A: The assembly of DNM can be performed in a PCR thermal cycler with a 0.1–0.3 °C/s ramp rate. Use the following thermal profile: 5 min at 97 °C, then 65 °C for 15 min, then 23 °C. For every step of lowering the temperature, use the ramp rate option of 0.1 ^o^C/s.

Solution B: Use slow gradual cooling by positioning the beaker right after boiling in a 50–55 °C environment and then, after an intermediate temperature stabilization, in the table.
